# A qualitative study of participants’ views on re-consent in a longitudinal biobank

**DOI:** 10.1186/s12910-017-0182-0

**Published:** 2017-03-23

**Authors:** Mary Dixon-Woods, David Kocman, Liz Brewster, Janet Willars, Graeme Laurie, Carolyn Tarrant

**Affiliations:** 1 Cambridge Centre for Health Services Research, University of Cambridge, Institute of Public Health, Forvie Site, Robinson Way, Cambridge, CB2 0SR UK; 2 Department of Health Sciences, Social Science Applied to Healthcare Research (SAPPHIRE) Group, University of Leicester, Leicester, UK; 3 Lancaster Medical School, Faculty of Health and Medicine, Lancaster University, Lancaster, LA1 4YW UK; 4 Department of Law, University of Edinburgh, Edinburgh, UK

**Keywords:** Biomedical research, Human subjects research, Informed consent, Research ethics, Social science research

## Abstract

**Background:**

Biomedical research increasingly relies on long-term studies involving use and re-use of biological samples and data stored in large repositories or “biobanks” over lengthy periods, often raising questions about whether and when a re-consenting process should be activated. We sought to investigate the views on re-consent of participants in a longitudinal biobank.

**Methods:**

We conducted a qualitative study involving interviews with 24 people who were participating in a longitudinal biobank. Their views were elicited using a semi-structured interview schedule and scenarios based on a hypothetical biobank. Data analysis was based on the constant comparative method.

**Results:**

What participants identified as requiring new consent was not a straightforward matter predictable by algorithms about the scope of the consent, but instead was contingent. They assessed whether proposed new research implied a fundamental alteration in the underlying character of the biobank and whether specific projects were within the scope of the original consent. What mattered most to them was that the cooperative bargain into which they had entered was maintained in good faith. They saw re-consent as one important safeguard in this bargain. In determining what required re-consent, they deployed two logics. First, they used a logic of boundaries, where they sought to detect any possible rupture with their existing framework of cooperation. Second, they used a logic of risk, where they assessed proposed research for any potential threats for them personally or the research endeavour. When they judged that a need for re-consent had been activated, participants saw the process as way of re-actualising and renewing the cooperative bargain.

**Conclusions:**

Participants’ perceptions of research as a process of mutual co-operation between volunteer and researcher were fundamental to their views on consent. Consenting arrangements for biobanks should respect the cooperative values that are important to participants, recognise the two logics used by research volunteers, and avoid rigidity. Agility may be favoured by tiered consent combined with strong oversight mechanisms; this approach requires evaluation.

## Background

Biomedical research increasingly relies on long-term cohorts and clinical registries involving use and re-use of biological samples and data stored in large repositories or “biobanks” over lengthy periods [1]. Such projects characteristically seek to create enduring resources for multiple future research endeavours that may not be specified fully in advance. Accordingly, the exact purposes to which data and samples might be put may not be fully anticipated at the outset: the precise studies to be undertaken may be defined over time, and may evolve in unforeseen ways as new research ideas, uses and possibilities are imagined and the available technologies, collaborations, sharing arrangements and analytic methods change. These longitudinal projects complicate traditional forms of consent to research that have tended (in practice at least) to treat consent as one-off, project-bound event where the aims and procedures of each individual study can be explicitly described up front [2–4].

Various approaches have been proposed in an effort to deal with the ethical dilemmas associated with biobanking consent [5]. They range from models that treat the initial consent as open-ended, unrestricted permission through to more dialogic, consultative models that visualise consent as an ongoing negotiated process between participants and researchers [6]. One widely used approach has been to obtain so-called “broad consent”, a term defined in different ways in the literature [7–9]. We use “broad consent” here to refer to initial consent that is both to biobank participation and also to multiple, as yet not fully defined, uses that are minimally restricted as long as they fall within a “framework for future research of certain types” [10]. Hansson and colleagues suggest that as long as “the information covers all the issues that are relevant for a person’s choice, then that person’s consent is appropriately informed”[7]. Though debate and contestation about the ethics of broad consent have persisted [4–6] a recent US National Institutes of Health (NIH) workshop endorsed broad consent [11], which is defined as “consent for an unspecified range of future research subject to a few content and/or process restrictions. Broad consent is less specific than consent for each use, but more narrow than open-ended permission without any limitations (i.e., “blanket” consent).” The statement identifies the benefits of broad consent, but also emphasises that it requires robust oversight, communication with donors, enforcement of standards, and evaluation. This position makes clear that broad consent is not a “free ride” for researchers: it is conditional on ongoing oversight (through, for example, ethics committees/institutional review boards and other mechanisms of governance) to provide safeguards for participants’ interests and other important values.

In this article, we focus specifically on one of these safeguards: that of re-consent. Re-consent can be defined as an action in which a research participant makes the decision to participate in a study once again, or consent to new elements of an existing study: “the process of seeking participant consent to change or update their existing consent to allow their samples and data to be used in a different way from that which was originally agreed” [3]. Re-consent can be readily distinguished from re-affirmation, where the intention is simply to express willingness to abide by a decision already made [12].

Situations where a re-consent process may be considered include those where an initial broad consent cannot defensibly be relied upon because of features of proposed future use are sufficiently novel or mark a significant departure from the terms on which the consent was originally given; where an initial specific consent does not cover a future intended use; where proposed modifications to projects appear to be beyond the scope of the original consent; where it is proposed to revisit a group of participants for whom a study has been dormant for some time, or when paediatric participants reach adulthood [13]. The data-sharing policies recently developed by large research funders are an example of where a re-consent process might, in principle, be triggered. For instance, the US National Institutes of Health (NIH) now require all researchers who receive NIH support to conduct genome-wide association studies to submit de-identified data to the database of Genotypes and Phenotypes (dbGAP), a centralised clearinghouse for study data. Data from many cohorts funded by NIH were established long before this rule was introduced, and thus did not anticipate sharing of data in this way.

Re-consent is not, however, always a straightforward solution [14]. Some of the difficulties are practical, and include possible large costs, problems in contacting research participants (for example, because of missing or incorrect contact information), and non-return of re-consent forms. These problems and others may result in the attrition of volunteers and the undermining of research activities [8, 15]. Also non-trivial is the potential for a re-consent process to trigger distress, anger, or other emotional reactions linked to perceptions of violations of privacy or breach of trust [12], the possibility that existing participants may withdraw consent altogether, and the risks that the integrity of a cohort could be undermined by having multiple consents for different purposes from different participants.

The question of what an ethically sound re-consent process would look like then arises. It clearly requires mechanisms for making decisions about when the limits of an original broad consent are reached as well as systems for ensuring that any procedures used to seek new consent are well-designed, operate efficiently, and are recognised by stakeholders as fair, reasonable, respectful, and attentive to their interests. The latter are especially important requirements given evidence that cooperation with the research enterprise is generally secured through participants’ beliefs in the wholesomeness and public value of the research endeavour, their trust in the institutions and individuals who recruit them as participants, and their expectations about how research is conducted and regulated [16–18]. Ensuring that a process recognized as legitimate is in place is also fundamental to ensuring the “social license” or legitimacy for the research enterprise more broadly [19, 20].

It is therefore especially critical that the basis of participants’ views on re-consent be well understood; insights into why people reason in the way they do is crucial to respecting their wishes [16, 21]. Yet, while participants’ views on broad consent have been studied extensively, much of the debate to date on re-consent has involved professional groups with a stake in these processes, such as researchers, ethicists and research managers [3]. A corpus of empirical studies is beginning to emerge that has examined the views of participants themselves on re-consent in areas such as cancer genetics research [22, 23] and twins research [24], but the literature remains small. How participants reason about re-consent in the context of longitudinal studies involving personal medical data and biological samples remains under-researched. In this paper we report a study that sought to investigate the views on re-consent of participants in a longitudinal biobank, with a particular interest in examining how people reason about when a need for re-consent should be triggered.

## Methods

Our study design was a qualitative interview study, for which ethical approval was obtained as well as consent from each participant (see further details at end of manuscript).

Semi-structured interviews were conducted with 24 participants in Generation Scotland, a biobank of human biological samples for medical research that has been funded since 2003 as a partnership between the Scottish University Medical Schools and the Scottish National Health Service (NHS). It includes over 30,000 volunteers who provide samples and data for research purposes and are followed over time [25]. Generation Scotland uses a model of broad consent combined with governance that was developed using a participatory process during its planning and recruitment phase by a scientific committee reporting to an Independent Scientific Advisory Board. The Generation Scotland Executive is now responsible for, inter alia, the governance arrangements, accountability to the public, the scientific rationale and scope within the broad consent given by participants, consideration of any relevant ethical issues, and the conditions under which researchers will be given access to derived data, project data and samples collected by Generation Scotland. An Access Committee reviews all applications to use Generation Scotland resources, with responsibility for ensuring that any application to conduct a project conforms to the consent and ethical approval obtained.

In our interviews we did not seek specifically to explore participants’ experiences and views of Generation Scotland, except insofar as we asked them to describe their recruitment to the resource. The main focus of the interviews was on using their experiences as long-term volunteers to consider scenarios based on a hypothetical biobank relating to heart disease, with the aim to determining under what circumstances they would view a re-consent process as necessary and what form it should take.

Only those who had previously agreed to be contacted about further studies by Generation Scotland were eligible for inclusion. In order to comply with data protection and ethical requirements, the recruitment process was managed by Generation Scotland. Invitational letters were sent by Generation Scotland to 90 volunteers. We stratified the sample according to responses to an exercise conducted by Generation Scotland where participants were asked for reconsent to transfer abroad of data and samples, contacting 37 who replied positively to this request, 37 who replied negatively, and 16 who did not reply to the request. The letters invited participants to take part in an interview study, and an enclosed information sheet explained that the study was being conducted by the University of Leicester and was confidential. Participants were invited to return a reply slip to the University of Leicester; respondents were contacted by a Leicester researcher. Those who agreed took part in a one- hour telephone interview that was digitally recorded, transcribed verbatim and fully anonymised. Informed consent was obtained from all participants.

The prompt guide used in the interviews was based on review of the literature and discussions within the project team and its collaborators. It sought to ensure some consistency and comparability across interviews, but was used flexibly in individual interviews. The first, very brief, part of the interview comprised questions about participants’ actual experiences in Generation Scotland (their motivations to participate, recollections of consent and re-consent processes). They were also asked in general terms about their views of an optimal re-consenting process most likely to safeguard their trust and wellbeing. Participants were not asked in detail about their responses to the re-consent exercise conducted by Generational Scotland, beyond asking them whether they remembered the exercise and, if they did, whether they had re-consented. In the second and more substantive part, participants were asked to discuss participation in a hypothetical biobank called “Heart of Scotland”.

Described to participants, the hypothetical project was modelled on Generation Scotland’s broad consent and governance model and other characteristics: it was run by a publicly-funded institution, collected data from people over time, and anonymised and securely stored all data. Unlike Generation Scotland, however, it focused solely on cardiac research, to enable exploration of people’s perceptions of the boundaries of projects. Participants’ views were sought on eight hypothetical scenarios that covered a range of possible alterations to the original consent, including changes of broad aims (e.g. extending beyond cardiac research to include cancer research), specific studies (e.g. involving access to new types of data) and changes to research governance (see Table 1). These scenarios were devised to include specific features of interest relating to re-consent; they were based on review of the literature and discussions within the research team and its collaborators to identify a range of exemplars of potentially contentious issues.

**Table 1:** Scenarios used in interviews

Scenario 1: Involvement of commercial company A new blood sample screening is invented by a commercial company. The inventors think this will make it possible to detect who is more susceptible to heart disease at a much earlier age than is now possible, but the device needs to be tested further before they can be sure. If it works, it is likely to make the company a lot of money. They would like to put an application in to use the Heart of Scotland data and samples to test it further.
Scenario 2: Brain research A new screening test is developed which the inventors think will make it possible to detect who is at risk of developing dementia at a very early age (30). At the moment there are no treatments that can fully prevent or treat dementia. It may be possible to support people better if they are known to be at risk, but it could also mean that people detected early live for a long time with unwanted knowledge and could suffer discrimination. The consent for Heart of Scotland does not mention diseases related to the brain; it only covers heart disease.
Scenario 3: Educational achievement and diet research Let’s say a group of researchers want to use the Heart of Scotland resources to study educational achievement and how it is affected by diet. There is no particular focus on heart disease, but the researchers think that the database can provide them with the answer they need without having to set up a whole new study.
Scenario 4: Cross checking with police records Heart of Scotland is now approached by other researchers. They are trying to do research on a particularly sensitive area relating to heart disease – they want to know if a history of crime in the family predicts outcomes of heart disease. They therefore want to check police records against Heart of Scotland data.
Scenario 5: Human embryos research Now, let’s imagine Heart of Scotland is approached by researchers wanting to use biological samples in research involving human embryos. In line with UK law, licensed research can only take place on embryos up to 14 days.
Scenario 6: Data sent overseas Heart of Scotland can allow access to anonymised data and samples to approved groups of researchers abroad.
Scenario 7: Feedback on personal health issues When Heart of Scotland was set up, it was made clear to participants that they would not get any feedback on clinical relevant findings. For example, if it was discovered that a person had a genetic mutation that meant that were at high risk of breast cancer, they would not be told by Heart of Scotland. A change in this policy is proposed.
Scenario 8: Cancer research Let’s imagine that Heart of Scotland has been running for 10 years. A change in funding means it is now being asked to use its samples and data to study cancer as well as heart disease.

Analysis of interview data was based on the constant comparative method [26]. This involved iteration between the developing analysis and data, supported by regular team discussions and sensitising constructs derived from the literature. The emerging categories were organised into a framework and used to code and index the transcripts using NVivo software. Categories were continually checked and modified to ensure an adequate ‘fit’ with the data, whilst also accounting for deviant cases.

## Results

Of the 90 Generation Scotland volunteers contacted, 24 consented to take part in this interview study (14 men and 10 women). Participants were between 32 and 81 years old, with a median age of 58 years. A quarter of participants were over 65 years. Participants’ occupational backgrounds were varied; just over half reported secondary level as their highest achieved educational attainment. Just over half (13) recalled the re-consent exercise conducted by Generation Scotland and all of these indicated that they had given re-consent. The remaining 11 did not remember the exercise or whether they had given re-consent.

### Motivations to participate

Consistent with the findings of previous research on biobanking, participants reported that the most powerful motivator for their volunteering both in the Generation Scotland project and the hypothetical Heart of Scotland project was the opportunity to contribute to the public good: participation was presented as altruistically-driven, and as offering, among other things, personal gratification or “warm glow”.

*You just want to be part of something, and if any way you can improve things, so anything like that, long term, it’s beneficial to other people, I tend to go for. (008)*

Some participants reported a generalised willingness to contribute to advancing medical knowledge and did not distinguish any particular beneficiaries; others focused on specific classes of beneficiaries (such as the people of Scotland).

*I just thought that it was a good thing to do […] to improve the quality of care and the early diagnosis of heart problems [and] basically to help your fellow man. (003)*

*It was looking at things about Scotland, and that was the main reason I would have done it […]. I wouldn’t have been so keen if it hadn’t been Scotland. (002)*

Beyond a sense of contributing to public good, some participants also identified the possibility of more personal benefit, for example expressing hope that their children and grandchildren could benefit from advances in medical knowledge. Some also referred to the health check and genetic testing undertaken as part of the research, which they assumed would offer personalised insights into their health.

*Because I do have heart problems, […] my father had heart problems, and there’s been various other members of family that, the upper generation that have had strokes and heart problems, so it would be of interest to me to see how I could have avoided it[…] It would probably benefit my children or their children. (001)*

*I think it would be a mixture; there’d be actually a few factors. One would be curiosity, two would be some kind of altruism in feeling that it might help research on heart conditions and thirdly there might be an element of self-interest in so far as, if all these things are being done to you maybe in the course of that something would crop up that would actually need to be looked at. (009)*

### Warrants of trust

The desire to contribute to the public good (or gain some personal health benefit, direct or indirect) was not unconditional. Participants sought warrants of trust to reassure them that enterprise in which they were being asked to participate was a wholesome one with the proper values and safeguards in place, and that they were not being gullible or exposing themselves to risk in agreeing to contribute. In this, the consent process had a critical practical and symbolic role.

*I remember being told it was confidential […] what was going to happen, and how that information would be used in the future. (002)*

*Having it clearly defined as to what is going to happen and what further things are going to happen, and what is not, certainly for me what will not happen. And being able to ask the question. (003)*

Though most (22 of 24) participants could remember few of the specific details in the consent process for Generation Scotland, they reported a strong sense of certainty that there must have been a proper consent process in place. They were adamant that the process would have been adequate, otherwise they would not have consented. Rather than the specifics of consent forms, what was important to the volunteers was their understanding of the cooperative bargain into which they had entered.

*It would have seemed very sensible and very confidential […] or I wouldn’t have signed it […] it didn’t stand out in my mind as anything that I needed to worry about. (007)*

Though they were clear that their consent was in support of a worthwhile endeavour and that the proper assurances about the conduct of the research were in place, they were equally clear that they did not see their consent to biobank participation as offering an “open ticket” to researchers to do whatever they wanted with the samples and data that had been volunteered. They did not, in general, regard themselves as having permanently relinquished all rights and interests in their data and samples.

*I didn’t think it would be used unless I gave my permission [for it] to be used, other than the procedures that I went through. (001)*

Without the right protections, some participants feared that what they understood as a relationship of cooperation with researchers might be jeopardised. For instance, they worried that researchers might, intentionally or unwittingly, stray beyond what participants understood as the acceptable limits to the scope of the research, leaving them feeling exploited, potentially damaging researchers’ reputations, inhibiting volunteers from taking part or causing existing participants to consider withdrawal.

*I think it’s very important you know when a piece of research like this is carried out that in no way is it a Trojan horse. It must not be - too often these things are launched and then things like such as this can be used in quite a knowing way, that leave ends that might have eluded or escaped the perception of the participants. Nevertheless it’s got to be true to […] its stated aims and purposes. [It] must be explicit and comprehensive and any kind of exploitation of the ignorance or innocence of the participants must be avoided at all costs. (009)*

### The logic of boundaries and re-consent: continuities and ruptures

One important way in which participants determined the acceptable limits of their consent was by assessing the extent to which proposed new research moved beyond, or sat within, their existing framework of cooperation. These judgements were grounded in what we dub *a logic of boundaries*. Where participants could identify continuities and alignments between the original consent and any newly proposed research, existing frameworks of cooperation remained intact; since the basis of their initial consent was still secure, re-consent was not judged to be required.

*I suppose it all comes back to the initial decision, that if you decide to participate in something then I wouldn’t have any objection really about any type of research being carried out using the data that was provided. It’s all one in the same thing, providing it for the initial study – or the reasons that I would have participated - would still apply, regardless of what type of research was being carried out. (Scenario 2: 006)*

Thus, when participants assessed new research as broadly within the boundaries of their original consent, they did not see that further permissions or re-consent would be needed for specific projects – though there was variability in individual responses as to what exactly would comprise the limits of these boundaries. For some participants, the addition of a new disease focus would not require additional consent, since they saw the original consent as enabling improvements in quality of life through research. Similarly, some participants identified continuity of the boundaries of their original consent where their motivations were to reduce risk of disease for future generations, or where they saw their protections (such as anonymization) remaining intact.

*I don’t think [re-consent] is required […] it’s fundamental actually to find that knowledge that if there is a risk of dementia then it should be treated, because people with dementia, they don’t have a life (Scenario 2: 021)*

*No I don’t have any difficulty with that at all, I don’t think there is any need to ask for that permission again (…) it’s obviously an important thing if you can affect how well or badly somebody, some of your youngsters are going to be affected then it’s worthwhile (Scenario 3: 004)*

*They should just go ahead. They shouldn’t need any permission, they should just go ahead, [it is still anonymised]. (Scenario 4: 014)*

Sometimes, however, participants identified ruptures between their frameworks of cooperation and proposed new research activities described in the scenarios. These discontinuities occurred when participants deemed the new elements of research to be overstepping the boundaries of the original agreement. These ruptures might occur in two ways: first, some fundamental change in the character of the biobank and the principles governing the cooperative bargain might be identified. Some participants saw such shifts as occurring, for example, when it was proposed to expand the remit of a biobank set up for heart research to include cancer and brain research, to allow data sent overseas as opposed to research being carried out in the UK, to widen access to data and samples to commercial companies rather than limiting it to publicly-funded research, or to extend into areas seen as not truly “medical” research.

*[To me] it’s a purely medical reason that they’re doing studies [and] I don’t see that the [new research] there is particularly medical. (Scenario 3: 019)*

A second way in which a rupture might occur was when a specific project was seen to be no longer covered by the original consent. Here, judgements were made about the extent to which the new project was “in scope”.

*I just think [diet and educational achievement is] a whole different thing. You know I think you’d need permission to do that. (Scenario 3: 005)*

A view that a new consent was needed – either to the nature of the biobank or for a specific project - was not necessarily associated with unwillingness to participate in the new research: it was instead an indication that the bargain between researchers and participants needed to be re-affirmed. For example, one of the scenarios used in the hypothetical Heart of Scotland project involved a commercial company’s involvement in research, though commercial involvement had been excluded from the original consent. Some participants expressed no anxieties about commercial interests, but still felt re-consent should be sought by researchers.

*The original consent said that there’d be no companies. […] So I think in that case if it was potentially going to a company who was likely to make a large profit, but could have the breakthrough, I think that should be separate permission. (Scenario 1: 015)*

### The logic of risk

A second way in which participants made judgements about whether re-consent might be required involved a *logic of risk*. When drawing on this logic, participants considered how far new research might be seen as ethically questionable – not just by them as individuals, but by broader publics – and thus pose risks to legitimacy. When participants drew on this logic, it dominated over all other considerations.

*I mean it only takes one person just to say oh, that’s not right, and then the whole sort of research thing could be in jeopardy […] if it came out that the data had been used for things that initially wasn’t asked for permission for, it could put everything else ‘all to cop’. (008)*

*That’s a hugely personal and emotional issue. […] Yeah, you imagine if someone was to be told that at the end of the day when they didn’t know they were involved in [human embryo research], I think some people might feel they’ve been exploited. (Scenario 5: 013)*

All hypothetical Heart of Scotland scenarios represented research issues that could potentially generate concerns, but participants varied in which they saw as activating logics of risk. The ‘riskiest’ scenario (the scenario that stimulated use of the logic of risk in the greatest number of participants) was one involving hypothetical access by researchers to police records, where 17 of the 24 participants reported that their reasons for requiring re-consent were rooted in judgements of risk. The least risky research scenario described hypothetical research comparing educational achievements and diet, with only four participants drawing on the logic of risk.

When the logic of risk was deployed, participants typically highlighted issues that they saw as requiring researchers to seek an endorsement from them as their partners. Participants were, for example, more likely to see as risky those scenarios that they judged as potentially breaching anonymity, which they saw as one of the key pillars of the cooperative bargain.

*Yes, [re-consent is] definitely [needed]. Again I think it’s encroaching on people’s private life rather than their health life. I think you would have to ask the particular person. (Scenario 4: 005)*

### Variability in orientations towards research cooperation and re-consenting

All participants were long-term volunteers in medical research and tended to approach the prospect of being asked to cooperate in new research positively; the logic of boundaries rather than the logic of risk mostly acted as their default logic, in part because of their faith in the warrants of trust governing the project.

*Maybe I’m naïve person, I don’t know. But I think if I’ve signed up to something then, unless it’s something horrific that is going to be terrible for mankind, I don’t need to be asked again and again for permission to use it. You’re a reputable organisation, otherwise you wouldn’t be doing any of this work, and therefore I shouldn’t have any fears as to how it’s going to be used. (Scenario 2: 004)*

However, individuals varied in their positions on different scenarios and the principles they used to arrive at those positions. No participant approached the hypothetical scenarios by consistently deploying either the logic of boundaries or the logic of risk only. Individual participants varied in how they used the two logics, how they balanced them, and what issues drew their attention in reaching judgements. No scenario was judged consistently across all participants. This suggests that no distinct ‘types’ of participants or consistency of response can be discerned. Although the hypothetical scenarios provided as part of the study deliberately involved elements of novelty, or issues deemed to be widely judged as ethically controversial, variations in participants’ responses indicated that no research scenario generated a monochromic response.

### Handling re-consent

Re-consent was described by participants as a critical element of the cooperative bargain. It was understood not only as offering an opting-out mechanism when research scenarios were judged unacceptable, but, perhaps more importantly, as a way to renew the foundations of cooperation.

*Absolutely, they must [ask for re-consent], yes. I think, this goes into the area of human rights, people’s rights and their liberty, absolutely, you know. (Scenario 4: 002)*

*I think that’s moving quite a bit away from the consent originally given […] so I would be happier in that situation to be asked to re-consent. (Scenario 2: 010)*

Participants offered a number of recommendations on feasible and acceptable consenting and re-consenting arrangements. They were explicit that practices of consent must retain meaning and purpose: for them, consent was an institutionalised space for the exercise of participants’ agency in the cooperative bargain. Accordingly, most participants (21 out of 24) emphasised the need for initial consent to be sound, and suggested ways in which the process could be improved, specifically by enabling participants to register individual choices. They felt that, offered a menu of possible consents (rather than a single broad consent), they could indicate both their preferences for the principle of research participation and any extensions or exceptions they might wish to make known at the outset. Participants understood such choices as ‘informed’ because they would be given information on what various types of research would and would not involve.

*I’m happy for other things, but maybe not that one, if you know what I mean. You could probably have – you know like you have with your donor card – you can have this, this or this, but not that. They can have my heart, they can have my liver, but they can’t have my eyes. So you can… possibly have a tick box to say yes, I can be used for this, can be used for that, but not for this, this or this. (017)*

Once the initial consent was in place, oversight mechanisms were seen as critical to ensuring the ongoing monitoring of the boundaries of the consent and of any risks.

*I think the oversight committee is a good thing to bring in because you can see that there is an authority observing the study and that any point they might say, well you’re stepping beyond the first consent. I think that’s good. (015)*

Participants perceived that oversight bodies would, where appropriate, make decisions on when new consents might be needed, but would not necessarily be enabled to authorise projects on their behalf. Particularly where the logic of risk was engaged, participants reported that new consent would still be required. Retaining the right to withdraw entirely was also identified as an important right to be safeguarded:

*Certainly if it’s going down the line of embryonic study and police stuff, no you need to contact the people. (003)*

*The most important thing to say is this whole process can be stopped and brought to a close and the information withdrawn or cancelled, as soon as the volunteer feels uncomfortable with it. I think that’s the most important thing, because I would not sign up if I felt for a second that I was joining a kind of unstoppable programme. I wouldn’t do it. I must know that I am free to stop (009)*

Participants offered suggestions on how the membership and functioning of oversight bodies might be optimised. They were especially keen that they included individuals who were themselves participants and had a stake in the outcome of decisions.

*It’s good to have a committee or whatever that’s there, that understands that sort of thing, and that says hang on a minute. And I think it’s good that a person that’s involved in the research can know that there’s, in the background there’s a committee that’s actually just making sure that everything is 100% proper. (004)*

*I think there probably has to be some legal input I would imagine, from some sort of legal background, ethics, you know, ethics, but I think participant members would be very useful. (002)*

## Discussion

A model of broad consent plus oversight remains popular among large-scale biobanks. Though some commentators have argued that broad consent is ethically unsound, participants in our study did not see it that way. Instead, they understood and accepted that it might not be possible to anticipate all possible uses of samples and data at the outset of a longitudinal study: what mattered most to them was that the cooperative bargain into which they had entered was maintained in good faith. The relevant question for them was therefore one of how to protect this bargain. Participants saw re-consent as one important safeguard, valued as a way of demonstrating respect for individuals’ values and preferences and as a means of securing the legitimacy of the research endeavour – its social licence - more broadly. What participants identified as requiring new consent was not, however, a simple matter predictable by algorithms about the scope of the consent, but instead was contingent. They assessed whether proposed new research implied a fundamental alteration in the underlying character of the biobank and whether specific projects were within the scope of the original consent. Participants used two distinct forms of reasoning in structuring their judgements about when and whether they should be asked to re-consent: the logic of boundaries, where they sought to detect any possible rupture with their existing framework of cooperation, and the logic of risk, where they assessed any potential threats for them personally or the research endeavour as a whole. Participants were keen to maintain the meaning behind consent as an ethical and purposeful practice, and proposed forms of tiered initial consent followed by active monitoring of need for re-consent as a way of assuring this status. When they judged that a need for re-consent had been activated, they saw the re-consent process as way of re-actualising and renewing the cooperative bargain between researchers and participants.

Our study does have some limitations. We had a response rate of 26% which, while not atypical of this kind of research, was not as high as we had hoped. Further, we had no way of determining the representativeness of our sample. Nonetheless, these findings are important for the debate on the arrangements for consent and re-consent, suggesting that no simple calculus can be constructed to specify categories of research where participants are likely to feel that re-consent is or is not be required. This finding is consistent with previous attempts to identify ‘controversial’ topics for which re-consent may be required [27]. For instance, categorising data use as ‘commercial’ or ‘non-commercial’ also does not predict acceptability [28–30]. Developing categories of types of participants is equally unlikely to be appropriate [31], and might lead to crises of representation that could weaken rather than strengthen the cooperative bargain. Clearly, however, some means of determining when re-consent is required is needed.

One answer might be to look to the law to provide the rules. But, though law will always have a role in providing a general framework of standards and enforcement, it cannot provide the answer in most cases where the possibility of re-consent arises [32]. Legal clarity on its own is unlikely to secure trust: as work on the social licence to operate has repeatedly demonstrated, going “beyond compliance” may be necessary for legitimacy [33]. Legal rules are also likely to introduce unnecessary rigidity. The very nature of the biobank enterprise over time — an inherently uncertain and unpredictable process — means that interpretations of the boundaries of original broad consent will always be a fluid matter. As Hoeyer argues, universal ‘solutions’ may be doomed to failure, and it may be time to acknowledge diversity and its implications [31].

Participants in our study instead proposed an approach that offered a form of informed flexibility. It would involve a two-stage regime. First, rather than a single broad consent, the initial consent would offer structured options that would enable participants to choose not to allow some uses of their sample and tissues from the outset. This kind of regime is consistent with what has been described in the literature as *tiered consent* [34]. It allows participants to indicate whether they consent to all future uses of their data and samples or to select from a menu of categorical options — for example, declining data-sharing outside a specific biobank. Previous studies have suggested that tiered initial consent appears to be an approach favoured by participants, providing a mechanism to respect individuals’ preferences without imposing undue burdens on researchers [34] – though not all agree [35].

A second stage would involve the possibility of consent either for fundamental changes to the character of the biobank or for specific projects deemed to be outside the scope of the original consent. Participants in our study were generally prepared to consider possible extensions to the scope of their consent, but they often, as previous studies have suggested, wanted to be asked and not merely informed [36]. To some extent, this second stage shares features with the model of dynamic consent proposed by Kaye and others [37]. Dynamic consent seeks to shift consent from a one-time, upfront process to one that runs through the life-time of the biobank, and is intended to create a more dialogic “ongoing relationship based on trust and reciprocity” [5]. However, the specifics of how such an approach would operate need considered attention [38]: the practicalities of managing dynamic consent are non-trivial, and how they can be managed in ways that ensure respect for and balance the interests of research participants, researchers, and society at large is not always clear [39]. Insisting that participants must consent anew to each study – as implied by some (though not all) models of dynamic consent - places considerable onus on participants to police the contours of their own consent; another risk is that it imposes a tyranny of choice, where participants are faced with information overload and risk of regret associated with having to choose between too many options [10]. Yet new consent to each proposed new use of samples and data did not seem to be what participants in our study sought: instead, they wanted new consent only when they perceived a breach in the terms of their original consent. A regime of agile consent that does not necessarily incorporate all features of the dynamic consent model might satisfy these requirements, and should be tested empirically in future studies.

What is clear is that oversight mechanisms to determine which studies appear to be candidates for a new consenting process, based on the kinds of reasoning that participants themselves use, will be required. Consistent with current practice in many biobanks, only those that appear to go beyond the scope of the original consent or to introduce risks would activate a new consenting process. However, in the model proposed by participants in our study, decision-making about whether new consent is required should assess not just the implications for individual participants but also the social licence for a particular biobank. Second, a critical feature of these oversight bodies is that they should, as participants in our study proposed, include participant representatives who can foster the kind of authentic research relationship where communication and dialogue have prime importance. Third, participants’ motivations should be fully respected, so that any decisions made should be accountable, justified, and clearly non-exploitative. For a voluntary endeavour such as research participation to thrive, the nature of the cooperative bargain to be struck must be recognised and honoured, and this is likely to require reciprocity and two-way communication over repeated encounters [3, 20].

## Conclusions

The question of how ethical obligations to research participants who have given broad consent to biosamples and medical records to be used for research can be safeguarded continues to attract attention. It is clear that no one-shot magic bullets, legal or otherwise, will secure the cooperative bargain that underpins participants’ engagement with biobanking research. Participants’ perceptions of research as a process of mutual co-operation between volunteer and researcher were fundamental to their views on consent. We found variation in how participants applied the logics of boundaries and of risk, suggesting that no calculus can readily be constructed to predict straightforwardly what might agitate concern. Our findings suggest that future consenting arrangements should be sensitive to the cooperative values that participants see as governing their relationships with longitudinal biobanks, recognise the two logics used by research volunteers, and avoid rigid models that assume monochromic responses. Views of participants in our study suggest that one model that would benefit from further evaluation would use flexible approaches, where, for example, tiered initial consent is combined with oversight mechanisms to ensure that possible new studies or alterations to the character of the biobank are given proper scrutiny, with participant involvement, to determine when new consent is needed.

## Abbreviations

dbGAP: Database of Genotypes and Phenotypes
NHS: National health service
NIH: US National institutes of health
